# Prodromal Dementia With Lewy Bodies: Clinical Characterization and Predictors of Progression

**DOI:** 10.1002/mds.27997

**Published:** 2020-02-11

**Authors:** Marleen van de Beek, Inger van Steenoven, Jessica J. van der Zande, Frederik Barkhof, Charlotte E. Teunissen, Wiesje M. van der Flier, Afina W. Lemstra

**Affiliations:** ^1^ Alzheimer Center Amsterdam, Department of Neurology, Amsterdam Neuroscience Vrije Universiteit Amsterdam Amsterdam University Medical Centers Amsterdam the Netherlands; ^2^ Department of Radiology and Nuclear Medicine Amsterdam Neuroscience Amsterdam University Medical Centers Amsterdam the Netherlands; ^3^ Institutes of Neurology and Healthcare Engineering University College London London England United Kingdom; ^4^ Neurochemistry Laboratory and Biobank, Department of Clinical Chemistry, Amsterdam Neuroscience Vrije Universiteit Amsterdam Amsterdam University Medical Centers Amsterdam the Netherlands; ^5^ Department of Epidemiology and Biostatistics Vrije Universiteit Amsterdam Amsterdam University Medical Centers Amsterdam the Netherlands

**Keywords:** Alzheimer's disease, dementia with Lewy bodies (DLB), Mild cognitive impairment (MCI)

## Abstract

**Objective:**

The objective of this study was to examine clinical characteristics, cognitive decline, and predictors for time to dementia in prodromal dementia with Lewy bodies with mild cognitive impairment (MCI‐LB) compared with prodromal Alzheimer's disease (MCI‐AD).

**Methods:**

We included 73 MCI‐LB patients (12% female; 68 ± 6 years; Mini Mental State Examination, 27 ± 2) and 124 MCI‐AD patients (48% female; 68 ± 7 years; Mini Mental State Examination, 27 ± 2) from the Amsterdam Dementia Cohort. Follow‐up was available for 61 MCI‐LB patients and all MCI‐AD patients (3 ± 2 years). We evaluated dementia with Lewy bodies core features, neuropsychiatric symptoms, caregiver burden (Zarit caregiver burden interview), MRI, apolipoprotein genotype, and cerebrospinal fluid biomarkers (tau/Aβ_1–42_ ratio). Longitudinal outcome measures included cognitive slopes (memory, attention, executive functions, and language and visuospatial functions) and time to dementia.

**Results:**

Parkinsonism was the most frequently present core feature in MCI‐LB (69%). MCI‐LB patients more often had neuropsychiatric symptoms and scored higher on ZARIT when compared with the MCI‐AD patients. Linear mixed models showed that at baseline, MCI‐LB patients performed worse on nonmemory cognitive domains, whereas memory performance was worse in MCI‐AD patients. Over time, MCI‐LB patients declined faster on attention, whereas MCI‐AD patients declined faster on the Mini Mental State Examination and memory. Cox proportional hazards regressions showed that in the MCI‐LB patients, lower attention (hazard ratio [HR] = 1.6; 95% confidence interval [CI], 1.1–2.3) and more posterior cortical atrophy (HR = 3.0; 95% CI, 1.5–5.8) predicted shorter time to dementia. In the MCI‐AD patients, worse performance on memory (HR = 1.1; 95% CI, 1.0–1.2) and executive functions (HR = 1.3; 95% CI, 1.0–1.6) were independently associated with time to Alzheimer's dementia.

**Conclusion:**

MCI‐LB patients have distinct neuropsychiatric and cognitive profiles with prominent decline in attention when compared with MCI‐AD patients. Our results highlight the importance of early diagnosis because symptoms already have an impact in the prodromal stages. © 2020 The Authors. *Movement Disorders* published by Wiley Periodicals, Inc. on behalf of International Parkinson and Movement Disorder Society.

Dementia with Lewy bodies (DLB) is the second most common form of neurodegenerative dementia in the elderly.^1^ DLB is characterized by cognitive impairment and core symptoms including fluctuating attention, hallucinations, parkinsonism, and rapid eye movement sleep behavior disorder (RBD).^2^ Prodromal symptoms in DLB can occur up to decades before the dementia phase.^3^, ^4^, ^5^ In Alzheimer's disease (AD), extensive research has been done on these prodromal stages, usually called mild cognitive impairment (MCI).^6^, ^7^ In contrast to MCI as a result of AD (MCI‐AD), research on MCI as a result of Lewy bodies (MCI‐LB) is limited.^4^

Some cross‐sectional studies suggest that the cognitive profile of MCI‐LB differs from MCI‐AD, with less pronounced memory impairment,^8^ but lower performance in attention,^3^, ^9^ executive,^8^, ^10^ and visuospatial functions.^10^, ^11^ Other studies in MCI‐LB found prominent neuropsychiatric symptoms (NPS), with frequent anxiety, apathy, and sleep disturbances.^3^, ^8^

Only a few longitudinal studies on MCI‐LB have been described. One study found that nonamnestic MCI patients most likely develop DLB, whereas amnestic MCI patients likely develop AD.^12^ Other studies found that imaging markers, such as magnetic resonance imaging (MRI), ^123^I‐FP‐CIT single‐photon emission computed tomography (SPECT) imaging, and EEG, can have prognostic value in the MCI stage.^13^, ^14^, ^15^

These former studies in MCI‐LB are limited by small sample sizes and/or cross‐sectional designs, therefore prognosis and disease course remain speculative at best. Here, we examined clinical characteristics, cognitive decline, and predictors of time to progression in MCI‐LB compared with MCI‐AD.

## Methods

### Study Population

We included 73 MCI‐LB patients and 124 MCI‐AD patients from the Amsterdam Dementia Cohort. All patients visited the memory clinic and underwent a 1‐day standardized diagnostic work‐up that included a semistructured medical history interview, informant‐based history, neurological and medical examinations, neuropsychological assessment, brain MRI, standard laboratory work‐up, and lumbar puncture.^16^ Diagnoses were made during a multidisciplinary meeting. Patients were invited for yearly follow‐up, which consisted of patient history, caregiver interview, standard physical examination, and neuropsychological assessment.

MCI is diagnosed according to the current diagnostic criteria, that is, concern reflecting changes in cognition, objective impairment in cognition, preservation of independence of functional abilities, and not demented.^17^ We selected MCI patients who had Mini Mental State Examination (MMSE) scores ≥25 and 1 impaired cognitive domain on extensive neuropsychological assessment. Inclusion criteria for MCI‐LB were (1) MCI and (2) at least 2 core clinical DLB features (visual hallucinations, parkinsonism, fluctuations, and/or RBD) at first presentation at our memory clinic (n = 57, 78%) or 1 clinical feature and abnormal ^123^I‐FP‐CIT SPECT (dopamine transporter (DAT)–SPECT; n = 6, 8%), and/or clinical diagnosis of probable DLB during follow‐up (n = 32, 44%). DAT–SPECT imaging was available in 47 MCI‐LB patients, of which 46 patients (98%) showed abnormal tracer uptake. MRI, cerebrospinal fluid (CSF) AD biomarkers (tau/Aβ_1–42_ ratio) and apolipoprotein (APOE)‐e4 status was available in most MCI‐LB patients (MRI, 79%; CSF, 70%; APOE‐ε4 status, 84%). Follow‐up neuropsychological assessment was available for 61 MCI‐LB patients (84%), and the average follow‐up time was 3.0 ± 2.0 years.

Inclusion criteria for MCI‐AD were MCI with abnormal AD CSF biomarkers (Aβ_1‐42_ < 813 pg/mL, tau >375pg/mL, p‐tau >52pg/mL, all abnormal). Patients had a last clinical diagnosis of MCI or probable AD at follow‐up. We selected patients who had CSF, MRI, APOE‐ε4 status, and at least a 1 year neuropsychological follow‐up available (average follow‐up time 3.0 ± 2.2 years). MCI‐AD patients were matched on age range with MCI‐LB patients. We used selection criteria for MCI‐LB and MCI‐AD that optimized diagnostic accuracy for both diseases. This resulted in selection criteria based on clinical features for MCI‐LB and selection criteria based on biomarkers for MCI‐AD.

All patients gave written informed consent for use of their clinical data. The local medical ethics committee of the Amsterdam University Medical Centers approved of the study.

### Outcome Measures

#### DLB Core Clinical Features

The presence of core clinical features was rated according to the McKeith 2017 criteria and was obtained from the standardized clinical work‐up (described previously).^2^ Parkinsonism was systematically assessed during the neurological exam and was rated present when the exam showed extrapyramidal signs (tremor, bradykinesia, and/or rigidity). The presence of hallucinations was systematically assessed with the informant‐rated Neuropsychiatric Inventory (NPI) and were scored as being present (NPI hallucinations score ≥ 1) or absent.^18^ The modality of the hallucinations (visual, tactile, acoustic) was obtained from the medical records. When the NPI hallucination score was not available, all information on hallucinations was obtained from medical records. Two raters independently reviewed the semistructured patient history interview for information on fluctuations and RBD. Fluctuations were rated positively when the patient or caregiver reported that the patients’ cognitive functioning fluctuated during the day and weeks. RBD was rated positively when caregivers reported that the patients seemed to “act out” their dreams and were moving extensively during sleep. Consensus was met between raters during a consensus meeting. Supplementary Figure S1 shows the distribution of the number of core clinical features at baseline.

#### Clinical Assessment

The Disability Assessment for Dementia is an informant‐rated questionnaire measuring the competence in instrumental activities in daily living (IADL), with scores ranging from 0% to 100%, and higher scores indicating higher competence.^19^ Neuropsychiatric symptoms were assessed with the 12‐item NPI, with information provided by the caregiver.^18^ We scored the symptoms of the NPI as being present (≥1) or absent. Depressive symptoms were assessed with the self‐reported Geriatric Depression Scale (GDS), with scores ranging from 0 to 15.^20^ The Zarit caregiver burden interview was used to assess caregiver burden, with scores ranging from 0 to 88. Scores between 0 and 20 indicate little to no burden, 21 to 40 indicate mild to moderate burden, and 41 to 88 indicate severe burden.^21^

#### Cognitive Assessment and Progression to Dementia

Cognition was assessed with a standardized test battery.^16^ The MMSE was used for assessing global cognition.^22^ Memory was tested using the Visual Association Test and the immediate recall and delayed recall of the Dutch version of the verbal learning test.^23^, ^24^ Attention and speed were measured with the Trail Making Test part A, Stroop Test parts 1 and 2, and the forward condition of the digit span (extended version).^25^, ^26^, ^27^ For executive functions, we used the score of the Trail Making Test part B controlled for the score on part A, the score of Stroop part 3 controlled for score on part 2, digit span backward (extended version), letter fluency, and the Frontal Assessment Battery.^26^, ^27^, ^28^, ^29^ We used 3 subtests of the Visual Object and Space Perception (VOSP) battery for visual–spatial functioning (number–location test, dot counting, and fragmented letters).^30^ Language was assessed with the Visual Association Naming Task and category fluency.^23^, ^28^ Missing individual neuropsychological test scores were imputed using multiple imputation of individual set scores. A total of 15 imputed datasets were created to ensure stability of the results. Analyses were done on the pooled datasets. Inverse scores were calculated for time‐dependent tests. Neuropsychological data were converted to *z* scores using the baseline data of an independent group of cognitively healthy subjects (n = 533, 60 ± 10 years, 54% female, MMSE = 29 ± 1). We created domain scores for attention, memory, executive function, language, and visuospatial functioning. Progression to dementia was defined as having impairment in 2 or more cognitive domains. Impairment in a cognitive domain was defined as having a domain score of *z* score < −2. All patients that progressed to dementia also fulfilled the clinical diagnosis for dementia, including interference in daily living.

### Biological Measures

CSF was obtained via lumbar puncture.^16^ CSF Aβ_1‐42_ and total tau concentrations were determined using Innotest (enzyme‐linked immunosorbent assay (ELISA); Innotest, Fujirebio, Gent, Belgium) or Elecsys Aβ_1–42_, tau, and p‐tau (181P) CSF assays (Roche Diagnostics, GmbH, Mannheim, Germany) run on the *cobas e*601 analyzer (Roche Diagnostics). Elecsys values of Aβ_1–42_ and tau were converted to Innotest values by using previously described formulas.^31^ We used the ratio total tau/Aβ_1–42_ > 0.52 to define concomitant AD pathology.^32^

MRI scanning was performed according to standardized protocol.^16^ Data were acquired using multiple scanners (1.5 and 3 Tesla)(Philips Medical Systems, Best, The Netherlands). Visual assessment of atrophy and cerebrovascular abnormalities was performed by experienced neuroradiologists.^16^ Medial temporal lobe atrophy was rated using coronal T1‐weighted images on a 5‐point scale (0–4).^33^ For the analysis, we used the average score of the left and right. Global cortical atrophy was rated on fluid attenuated inversion recovery images using a 4‐point scale (0–3).^34^ Posterior cortical atrophy (PCA) was rated on T1‐weighted and fluid attenuated inversion recovery weighted images in sagittal, axial, and coronal planes, with an average of the left and right scores (range 0–3).^35^ White matter hyperintensities were rated on axial fluid attenuated inversion recovery (FLAIR) images using the Fazekas scale (range 0–3).^36^ The number of microbleeds was dichotomized as present or not (0–1).^16^, ^37^

The APOE‐ε4 genotype was determined using the LightCycler ApoE mutation Detection Kit (Roche Diagnostics) after DNA isolation from 10 mL ethylenediamine tetraacetic acid (EDTA) vacutainer tubes. Based on the APOE‐ε4 genotype, patients were classified as APOE‐ε4 carriers (heterozygous and homozygous) or noncarriers.

Orthostatic hypotension was defined as a 20 mmHg drop in systolic blood pressure or a 10 mmHG drop in diastolic blood pressure between supine and standing positions.

### Statistical Analysis

Analyses were performed using Statistical Package for the Social Sciences (version 22, IBM, Armonk, NY) and R (version 3.2.5, R Development Core Team, Vienna, Austria). To assess group differences at baseline, *t* tests, Mann‐Whitney *U* tests, and χ^2^ were used depending on normality. To assess the course of cognitive decline between diagnostic groups, linear mixed models were used. The model included time (in years), diagnosis and the interaction between time and diagnosis for the different cognitive domains as dependent variables, for example, MMSE, memory, attention, executive functioning, language, and visuospatial functioning. The model included a random intercept. The MCI‐AD patients were used as a reference group. β + standard error (SE) for diagnosis represents the difference in baseline cognitive domain scores for MCI‐LB. β + SE for the interaction between time and diagnosis represents the difference in annual decline in cognitive domain for MCI‐LB. Finally, we used Cox proportional hazard analyses to identify predictors for time to dementia. First, we performed univariate age‐adjusted models (model 1), evaluating sex, cognitive scores, core clinical features, GDS, CSF tau/Aβ_1–42_ ratio, and MRI markers as putative predictors. Subsequently, we constructed a multivariate model (model 2) with the significant predictors of the univariate models using backward modeling. Cox proportional hazards analyses were performed separately for the MCI‐LB and MCI‐AD patients to provide a comparison of predictors. For visualization, we constructed Kaplan‐Meier curves of the significant predictors in MCI‐LB. We dichotomized the variables using predefined cut‐offs. When no cut‐offs were available, we used the split‐half approach to dichotomize the variables using the median value as a cut‐off point.

## Results

**Figure 1:**
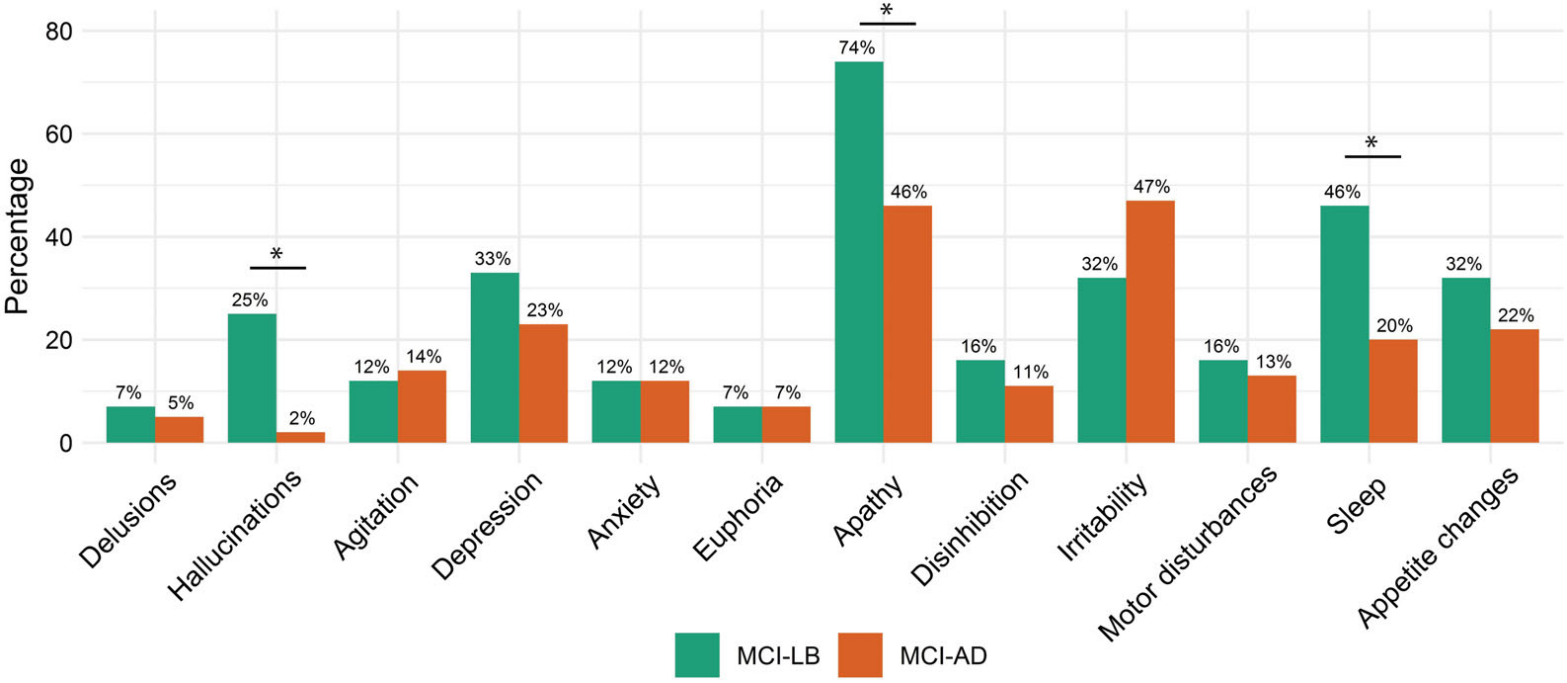
The prevalence of Neuropsychiatric Inventory (NPI) symptoms in MCI‐LB (n = 57) and MCI‐AD (n = 104). *P < 0.05, χ^2^ test. MCI‐AD, Alzheimer's disease with mild cognitive impairment; MCI‐LB, dementia with Lewy bodies with mild cognitive impairment. [Color figure can be viewed at wileyonlinelibrary.com]

The demographic and clinical data are presented in Table 1. Parkinsonism was the most frequent core clinical feature in MCI‐LB (70%), whereas hallucinations, fluctuations, and RBD were reported in about half of the patients (51%, 51%, and 47%, respectively). The MCI‐LB group had more men compared with the MCI‐AD group (*P* < 0.001). Age, years of education, and disease duration did not differ between the MCI‐LB and MCI‐AD groups. No differences were found on IADL dependency. The caregiver‐rated NPI indicated more NPS in the MCI‐LB patients, in particular apathy, which was more frequently noted in the MCI‐LB patients compared with the MCI‐AD patients (74% vs. 46%). As expected, based on our selection criteria, hallucinations and sleep disturbances were more frequently reported by caregivers of MCI‐LB patients (Fig. 1). The MCI‐LB patients scored higher on the GDS than the MCI‐AD patients, indicating more depressive symptoms. The Zarit caregiver burden questionnaire indicated higher caregiver burden for MCI‐LB than MCI‐AD.

**Table 1:** Baseline characteristics

Variable	MCI‐LB	n	MCI‐AD	n
Sex, female, n (%)	9 (12)^a^	73	60 (48)	124
Age, mean ± SD	67.9 ± 6.1	73	68.0 ± 6.6	124
Years of education, mean ± SD	12 ± 3	73	12 ± 3	124
Disease duration, y, mean ± SD	4 ± 4	72	3 ± 2	124
MMSE, mean ± SD	27 ± 2	73	27 ± 2	124
Follow‐up time, mean ± SD	3.0 ± 2.0	61	3.0 ± 2.2	124
DLB core features, n (%)				
Visual hallucinations	37 (51)	73	0	124
Parkinsonism	51 (70)	73	2 (2)	124
Fluctuations	37 (51)	73	3 (2)	124
RBD	34 (47)	73	1 (1)	124
Abnormal DAT‐SPECT	46 (98)	47		
Questionnaires, median (IQR)				
GDS	3 (2–5)^a^	62	2 (1–4)	104
DAD	90 (74–97)	46	95 (85–100)	55
Zarit caregiver burden interview	22 (11–32)^a^	28	12 (3–20)	17
NPI total score	10 (6–16)^a^	57	5 (1–10)	104
CSF T‐tau/Aβ_1–42_ ratio > 0.52, n (%)	18 (35)^a^	51	100	124
APOE‐ε4 carrier, n (%)	29 (48)^a^	61	100 (81)	124
Orthostatic hypotension, n (%)	20 (43)	47	14 (26)	54
MRI characteristics, median (IQR)				
Medial temporal atrophy	1 (0–1)	58	1 (0–2)	111
Global cortical atrophy	1 (0–1)	57	1 (0–1)	111
Posterior cortical atrophy	1 (1–2)	54	1 (1–1)	107
WMH, Fazekas	1 (1–1)	54	1 (0–1)	111
Microbleeds, presence, n (%)	12 (25)	48	29 (27)	109


a *P* < 0.05 compared to MCI‐AD.

Patients were matched on age range. MCI‐AD patients were selected based on having abnormal CSF biomarkers.

MCI‐LB, mild cognitive impairment due to Lewy Bodies; MCI‐AD, mild cognitive impairment due to Alzheimer's diseaset; MMSE, Mini Mental State Examination; DLB, dementia with Lewy bodies; RBD, rapid eye movement sleep behavior disorder; DAT‐SPECT, dopamine transporter with single‐photon emission computed tomography; IQR, interquartile range; GDS, Geriatric Depression Scale (15 items); DAD, Disability Assessment for Dementia; NPI, Neuropsychiatric Inventory; CSF, cerebrospinal fluid; APOE, apolipoprotein; MRI, magnetic resonance imaging; WMH, white matter hyperintensities.

Orthostatic hypotension was present in 43% of the MCI‐LB patients compared with 26% of the MCI‐AD patients (*P* > 0.05*)*. There were no differences in MRI characteristics between groups. The MCI‐LB patients were less often APOE‐ε4 carriers when compared with the MCI‐AD patients. Of the MCI‐LB patients, 35% had CSF profiles compatible with AD at baseline, whereas by definition, all MCI‐AD patients had abnormal CSF biomarker profiles.

### Cognitive Assessment and Progression to Dementia

**Figure 2:**
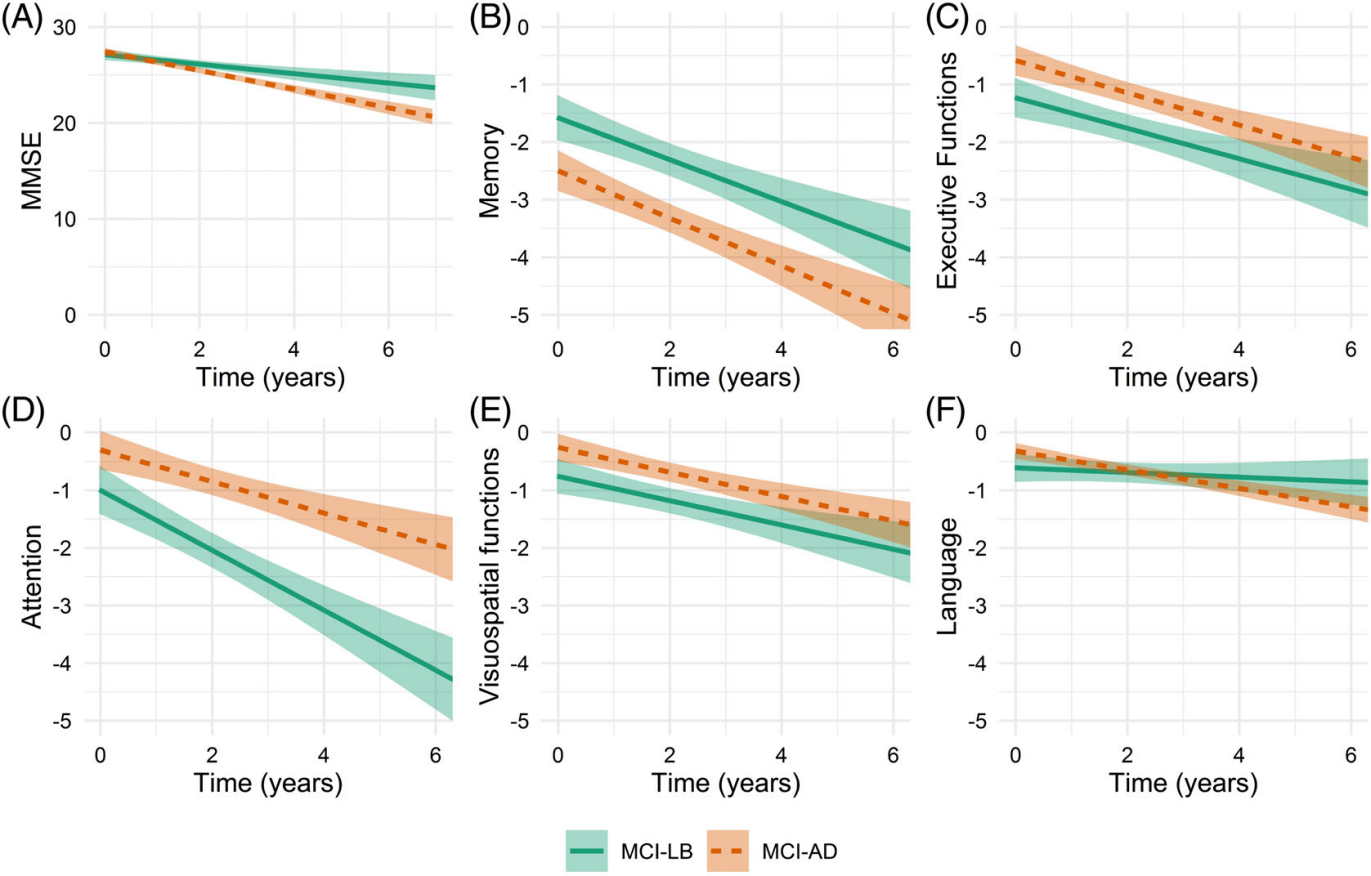
Estimated trajectories of cognitive domains. Regression lines represent estimated group trajectories over time in years with 95% confidence intervals based on nonimputed data. (**A**) MMSE score (range 0–30). (**B–F**) Data represent *z* scores based on cognitively healthy subjects. MCI‐AD, mild cognitive impairment due to Alzheimer's disease; MCI‐LB, mild cognitive impairment due to Lewy Bodies; MMSE, Mini Mental State Examination. [Color figure can be viewed at wileyonlinelibrary.com]

Linear mixed models, adjusted for age, sex, and education, were used to assess the course of cognitive decline (Supplementary Table S1, Fig. 2). At baseline, the MCI‐LB patients performed worse on executive and visuospatial functioning when compared with the MCI‐AD patients. Over time, the MCI‐LB patients declined faster on attention. By contrast, the MCI‐AD patients had lower baseline memory and had steeper declines over time in this domain when compared with the MCI‐LB patients. In addition, the MCI‐AD patients declined faster on the MMSE.

Of the 61 MCI‐LB patients with follow‐up available, 32 (53%) progressed to dementia during follow‐up after an average of 2.7 ± 1.7 years. In the MCI‐AD group, 85 of 124 patients (69%) progressed to dementia at follow‐up after 2.6 ± 1.7 years. The progression rate and time to dementia did not differ between the MCI‐LB and MCI‐AD patients.

**Figure 3:**
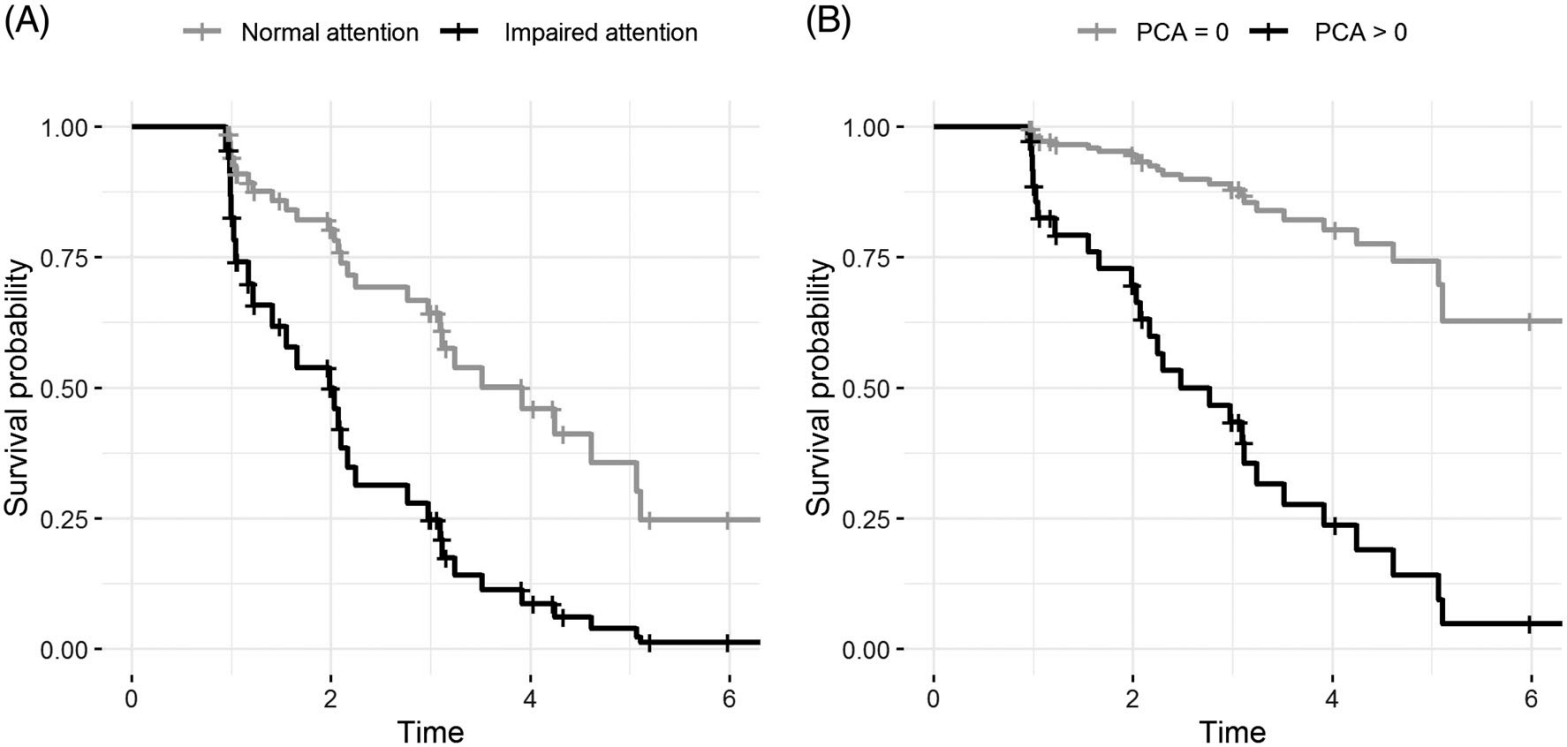
Survival curves for predictors of progression to dementia in mild cognitive impairment due to Lewy Bodies: attention (**A**) and PCA (**B**). For attention, a *z* score < −2 was considered impaired. For PCA, we used the median value as a cutoff (1). PCA, posterior cortical atrophy.

Age‐adjusted Cox regression analyses showed that lower scores on memory, attention, and MMSE were associated with decreased time to dementia in MCI‐LB patients. In addition, higher PCA scores and higher GCA scores were associated with shorter time to dementia (Fig. 3, Table 2). There were no associations with age, sex, core clinical features, GDS, T‐tau/Aβ_1–42_ ratio, or APOE‐ε4. Subsequently, in the multivariate model, PCA and attention remained independent predictors of progression to dementia (Table 2). In patients with MCI‐AD, higher medial temporal lobe atrophy scores and lower scores on memory, attention, and executive functions were related to shorter time to dementia. In the multivariate model, memory and executive functions remained independent predictors of progression to AD (Table 2).

**Table 2:** Cox proportional hazards regressions used to determine predictors for time to progression to dementia in MCI‐LB and MCI‐AD

	MCI‐LB		MCI‐AD	
	Model 1	Model 2	Model 1	Model 2
Age	1.0 (1.0–1.1)		1.0 (1.0–1.0)	
Sex	1.1 (0.3–3.8)		1.0 (0.6–1.5)	
MMSE^a^	**1.4 (1.1–1.8)**		1.1 (1.0–1.3)	
Memory^a^	**1.3 (1.0–1.7)**		**1.1 (1.0–1.2)**	**1.1 (1.0–1.2)**
Attention^a^	**1.5 (1.1–2.0)**	**1.6 (1.1–2.3)**	**1.2 (1.0–1.5)**	
Executive functions^a^	1.3 (1.0–1.6)		**1.2 (1.0–1.5)**	**1.3 (1.0–1.6)**
GDS	1.1 (0.9–1.3)		1.1 (0.9–1.2)	
Orthostatic hypotension	0.6 (0.2–1.6)		0.9 (0.4–2.3)	
APOE‐e4 carrier	1.1 (0.5–2.5)		1.0 (0.5–1.8)	
Medial temporal atrophy	**1.5 (0.9–2.4)**		**1.3 (1.0–1.8)**	
Posterior temporal atrophy	**3.2 (1.6–6.2)**	**3.0 (1.5–5.8)**	1.0 (0.7–1.5)	
Global cortical atrophy	**2.3 (1.2–4.3)**		1.5 (1.0–2.4)	
CSF T‐tau/Aβ_1–42_ ratio	1.1 (0.4–3.0)			
Number of features	1.1 (0.8–1.5)			
Hallucinations	0.7 (0.3–1.4)			
Parkinsonism	1.3 (0.7–2.9)			
Fluctuations	0.6 (0.3–1.2)			
RBD	1.3 (0.6–2.7)			

a Because a lower score indicates worse performance, these scores were inverted.

Data represent hazard ratio (95% confidence interval). Cox proportional hazards regressions include putative predictors as independent variables and progression and time to dementia as outcome variable. Model 1: univariate associations. Model 2: multivariate associations with after backward selection with significant predictors of Model 1. Bold hazard ratios and 95% confidence intervals depict significant associations (*P* < 0.05).

MCI‐LB, mild cognitive impairment due to Lewy Bodies; MCI‐AD, mild cognitive impairment due to Alzheimer's disease; MMSE, Mini Mental State Examination; GDS, geriatric depression scale; APOE, apolipoprotein; CSF, cerebrospinal fluid; RBD, rapid eye movement sleep behavior disorder.

## Discussion

In this study, we assessed clinical characteristics, cognitive decline, and predictors for time to dementia in a large cohort of MCI‐LB patients compared with MCI‐AD patients. First, we found that parkinsonism was present in 70% of the MCI‐LB patients, whereas other core features (hallucinations, cognitive fluctuations, and RBD) were reported in roughly half of the MCI‐LB patients. Second, we found that the MCI‐LB patients had more NPS, in particular apathy and depressive symptoms, and that they had distinct cognitive profiles with prominent declines in attention when compared with the MCI‐AD patients. Finally, lower attentional function and more posterior cortical atrophy at first visit were independent predictors of time to dementia in MCI‐LB, whereas in MCI‐AD lower memory and executive functions were predictive of time to dementia.

Consistent with previous studies in established DLB, we found more noncognitive symptoms and higher caregiver burden in MCI‐LB compared with the MCI‐AD.^38^, ^39^ Noncognitive symptoms have previously been related to higher caregiver burden, lower quality of life, and earlier nursing home admission in DLB.^39^, ^40^, ^41^ Our results show that already in the MCI phase, noncognitive symptoms contribute to caregiver burden. This finding is important as noncognitive symptoms are potentially treatable.^42^ Early diagnosis is mandatory because it can help in managing symptoms and reducing the impact on patients and caregivers.

The cognitive profile in MCI‐LB differs from (MCI‐)AD, with less severe memory impairment and more executive and visuospatial impairment.^43^ This cognitive profile is in accordance with previous cross‐sectional studies investigating MCI‐LB.^3^, ^8^ Over time, the MCI‐LB patients had steeper declines in attention, and they declined less on memory compared with the MCI‐AD patients.

The progression rate and time to dementia did not differ between MCI‐LB and MCI‐AD. One other study investigated the progression to dementia in MCI‐LB and MCI‐AD and found similar progression rates and time to dementia.^12^ Although the progression rate was comparable between MCI‐LB and MCI‐AD, the predictors of time to dementia were different between these diseases. For MCI‐AD, lower memory and executive functions were associated with shorter time to Alzheimer's dementia, consistent with findings from previous studies.^44^, ^45^ In contrast, in MCI‐LB, lower attention at first visit was associated with faster progression to dementia. This finding seems intuitive because DLB is characterized by prominent attention dysfunction early in the disease course. The MCI‐LB patients with lower attention might be closer to the dementia phase than patients with relative sparing of these functions.

In addition, cortical atrophy in MCI‐LB was associated with swifter progression to dementia. Specifically, higher GCA scores and higher PCA scores were more commonly seen in patients who progressed to DLB, and they were determinants of progression to dementia. The prognostic value of these atrophy scales seems specific for MCI‐LB patients, as we did not find any prognostic value of GCA or PCA for MCI‐AD patients. Recent studies showed that GCA and PCA can occur in DLB patients, independent of amyloid pathology, but are likely related to tau pathology and α‐synuclein pathology.^46^, ^47^, ^48^ This suggests that there is a DLB‐specific process that is related to these atrophy patterns. Our results add to the importance of these markers, as they seem to be related to progression in an early stage of the disease.

Core clinical features and CSF AD biomarkers did not predispose for rapid progression to dementia. Neither the separate core clinical features nor the amount of core features that were present at first visit predicted time to dementia. In addition, although concomitant AD pathology has previously been related to worse prognosis in the dementia phase, CSF AD biomarkers did not predict time to dementia in MCI‐LB.^49^ In addition, our results show fewer patients with concomitant AD pathology compared to studies in autopsy‐confirmed DLB patients.^50^ This indicates that the prognostic value of CSF AD biomarkers is poor in an early stage of the disease, and it is possible that concomitant AD pathology develops in later stages of the disease. Future longitudinal studies should elude on this topic.

There are several strengths in the present study, of which most notably the large and well‐defined cohort of MCI‐LB patients and the longitudinal design. Possible limitations entail the retrospective nature of the study. Missing values of core clinical features were rated retrospectively based on information in the medical charts. With this method, there is some risk of information and recall bias. Also, the retrospective design limits us to the use of rating core clinical features as being present or not, and the severity of symptoms is left out. Future prospective studies should use standardized methods to assess symptom severity. Second, we defined the moment of progression to dementia based on decline on cognitive tests, and functional decline was left out. It is important to note that our patients were followed up in a clinical setting and that clinicians diagnosed the patients with dementia as well, therefore the patients also fulfilled the criterion for interference in daily living based on clinical decision making. Yet the moment of progression was determined by cognitive scores, which could potentially introduce bias. For future studies, it would be interesting to look at progression in a broader sense by taking into account decline in IADL functioning or progression of NPS. Third, not all patients had sufficient follow‐up available, and therefore not all patients had progressed to dementia. It is possible that patients who are still in the MCI phase progress to a different type of dementia than expected based on our selection criteria. To accurately estimate prognosis, longer follow‐up duration is necessary.

Our findings underline the importance of timely diagnosis. Our results show that impairment in MCI‐LB is not restricted to cognition, but there is broad symptomatology that affects patients as well as caregivers. Some of these noncognitive symptoms occur even prior to the onset of cognitive symptoms. The term *MCI‐LB* could be insufficient in capturing patients in these earliest stages of DLB because it still focuses on cognitive impairment. A diagnosis of prodromal DLB should change to a classification that is not as much focused on cognitive staging. For the sake of both adequate patient management as well as shortening the time of insecurity for patients and their relatives, early diagnosis is mandatory. There is an urgent need for specific biomarkers, and a new set of diagnostic criteria should be considered in which cognitive impairment is not necessarily leading. Analogs could be found in diseases as Huntington's disease or progressive supranuclear palsy in which a certain constellation of symptoms can lead to a high probability diagnosis.

In summary, MCI‐LB patients have distinct symptoms, cognitive trajectories, and predictors of progression when compared with MCI‐AD patients. Further studies should elucidate whether early treatment has an impact on disease progression.

## Author Roles

(1) Research project: A. Conception, B. Organization, C. Execution; (2) Statistical Analysis: A. Design, B. Execution, C. Review and Critique; (3) Manuscript: A. Writing of the first draft, B. Review and Critique.

M.v.d.B.: 1A, 1B, 1C, 2A, 2B, 2C, 3A

I.v.S.: 1A, 1B, 1C, 2C, 3B

J.v.d.Z.: 2C, 3B

F.B.: 2C, 3B

C.T.: 2C, 3B

W.v.d.F.: 1C, 2C, 3B

A.L.: 1C, 2C, 3B

## Financial Disclosures of all authors (for the preceding 12 months)

Dr. Barkhof is supported by the NIHR biomedical research centre at UCLH. Research of Dr. Teunissen is supported by the European Commission (Marie Curie International TrainingNetwork, JPND), Health Holland, the Dutch Research Council (ZonMW), The WestonBrain Institute, Alzheimer Netherlands. Dr. Teunissen has a collaboration contract with ADx Neurosciences, performedcontract research or received grants from Probiodrug, AC Immune, Biogen‐Esai,CogRx, Toyama, Janssen prevention center, Boehringer, AxonNeurosciences,Fujirebio, EIP farma, PeopleBio, Roche. Research programs of Dr. van der Flier have been funded by ZonMW, NWO,EU‐JPND, Alzheimer Nederland, CardioVascular Onderzoek Nederland, Health~Holland, Topsector Life Sciences & Health, stichting Dioraphte, Gieskes‐Strijbisfonds, stichting Equilibrio, Biogen MA Inc, Life‐MI, AVID, Combinostics. Dr. van der Flier holds the Pasman chair. Dr. van der Flier has performed contract research for Biogen MA Inc. All funding is paid to her insitution. Dr. Lemstra has received funding from stichting Dioraphte and ZonMW Memorabel (project#733050509).The remaining authors report no actual or potential financial disclosures.

## Supporting information


**Supplementary Table 1** Estimated baseline cognition and change over time

**Supplementary Figure 1** Number of core features present in MCI‐LB (n = 73).
