# Supplementary material for: Prodromal Dementia With Lewy Bodies: Clinical Characterization and Predictors of Progression
Source: Mov Disord. 2020 Feb 11;35(5):859–67. doi: 10.1002/mds.27997 (PMC7317511; doi:10.1002/mds.27997)
Supplement: Supplementary file 1 — Supplementary Table 1 Estimated baseline cognition and change over time [file MDS-35-859-s001.docx]

| **Supplementary table 1** \| Estimated baseline cognition and change over time | | |
| --- | --- | --- |
|  | **MCI-LB vs MCI-AD** | |
|  | Estimated effect on baseline | Estimated change over time |
| **MMSE** | -.07 ± .41 | **.39 ± .12*** |
| **Memory** | **.86** ± **.27*** | **.12 ± .07**^+^ |
| **Attention** | -.49 ± .31 | **-.27 ± .10*** |
| **Executive Functions** | **-.49 ± .16*** | .00 ±.05 |
| **Visuospatial Functions** | **-.53 ± .24*** | -.04 ± .08 |
| **Language** | -.16 ± .13 | .05 ± .05 |
| Data are represented as β±SE, using pooled data of 15 imputed datasets. Negative betas represent a lower score for MCI-LB compared to MCI-AD at baseline, or steeper decline compared to MCI-AD over time. **p* ≤ 0.05, ^+^*p* ≤ 0.1. | | |
