# Supplementary figures and images for: Prodromal Dementia With Lewy Bodies: Clinical Characterization and Predictors of Progression
Source: Mov Disord. 2020 Feb 11;35(5):859–67. doi: 10.1002/mds.27997 (PMC7317511; doi:10.1002/mds.27997)

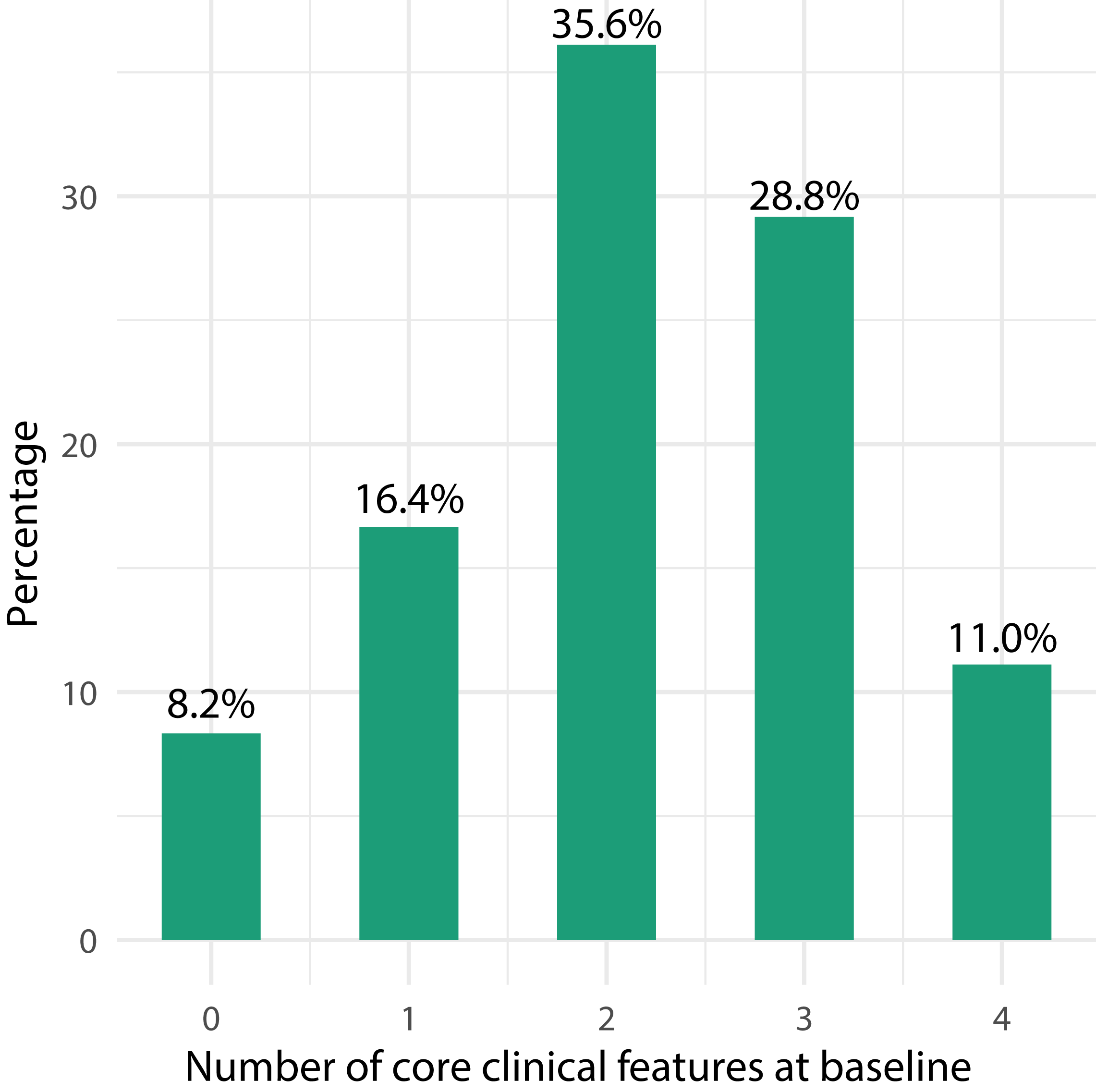

Supplement: Supplementary file 2 — Supplementary Figure 1 Number of core features present in MCI‐LB (n = 73). [file MDS-35-859-s002.tif]
